# Protocol for a pragmatic trial of Cannabidiol (CBD) to improve chronic pain symptoms among United States Veterans

**DOI:** 10.1186/s12906-024-04558-3

**Published:** 2024-06-29

**Authors:** Rachel S. Bergmans, Riley Wegryn-Jones, Catherine Klida, Vivian Kurtz, Laura Thomas, David A. Williams, Daniel J. Clauw, Kelley M. Kidwell, Amy S. B. Bohnert, Kevin F. Boehnke

**Affiliations:** 1 Medical School, Department of Anesthesiology, Chronic Pain and Fatigue Research Center, University of Michigan, Ann Arbor, USA; 2 Medical School, Department of Anesthesiology, University of Michigan, Ann Arbor, USA; 3 School of Public Health, Biostatistics, University of Michigan, Ann Arbor, USA; 4 Department of Veterans Affairs, Health Services Research & Development (HSR&D), Center for Clinical Management Research, Ann Arbor, USA; 5 Medical School, Department of Anesthesiology, University of Michigan, Michigan Psychedelic Center, Ann Arbor, USA

**Keywords:** Cannabis, Cannabinoids, Marijuana, Community-engaged research, Veteran health, Analgesic, Pain, Clinical Trial, Randomized Controlled Trial

## Abstract

**Background:**

Chronic pain affects over 100 million Americans, with a disproportionately high number being Veterans. Chronic pain is often difficult to treat and responds variably to medications, with many providing minimal relief or having adverse side effects that preclude use. Cannabidiol (CBD) has emerged as a potential treatment for chronic pain, yet research in this area remains limited, with few studies examining CBD’s analgesic potential. Because Veterans have a high need for improved pain care, we designed a clinical trial to investigate CBD’s effectiveness in managing chronic pain symptoms among Veterans. We aim to determine whether CBD oral solution compared to placebo study medication is associated with greater improvement in the Patient Global Impression of Change (PGIC).

**Methods:**

We designed a randomized, double-blind, placebo-controlled, pragmatic clinical trial with 468 participants. Participants will be randomly assigned in a 1:1 ratio to receive either placebo or a CBD oral solution over a 4-week period. The trial is remote via a smartphone app and by shipping study materials, including study medication, to participants. We will compare the difference in PGIC between the CBD and placebo group after four weeks and impacts on secondary outcomes (e.g., pain severity, pain interference, anxiety, suicide ideation, and sleep disturbance).

**Discussion:**

Once complete, this trial will be among the largest to date investigating the efficacy of CBD for chronic pain. Findings from this clinical trial will contribute to a greater knowledge of CBD’s analgesic potential and guide further research. Given the relative availability of CBD, our findings will help elucidate the potential of an accessible option for helping to manage chronic pain among Veterans.

**Trial registration:**

This protocol is registered at clinicaltrials.gov under study number NCT06213233.

## Background

Over 100 million Americans have chronic pain, which costs over $500 billion annually in health care and lost productivity [1]. Chronic pain is especially common among Veterans, affecting 25–30% of Veterans relative to 17–21% of the general population [2–4]. Unfortunately, many standard treatment protocols for chronic pain include pharmacological therapies that often provide inadequate pain relief or have adverse effects that can outweigh the benefits [5, 6].

Some of the challenges in chronic pain treatment stem from the high variability in symptoms and the various mechanisms that influence the experience of pain across individuals. Currently there are three recognized categories of pain mechanisms which can be present singularly or simultaneously with differential responses to pain treatments [7–11]. Nociceptive pain is due to tissue damage or inflammation; neuropathic pain is due to nerve damage or entrapment; and nociplastic pain is due to heightened sensitization in the central nervous system that can augment and maintain pain despite a lack of tissue or nerve damage [10, 12]. Personalized pain medicine, where suspected underlying pain mechanisms inform treatment selection, is largely unexplored for many analgesics, including compounds found in cannabis such as cannabidiol (CBD).

CBD is increasingly recognized for its anti-inflammatory and analgesic properties with rare or minor adverse effects [13–17]. However, CBD is yet to be effectively integrated within clinical care standards for treating chronic pain. This is partly attributable to the limitations of existing evidence. Among previous randomized controlled trails (RCTs) of CBD for pain, the results are inconsistent; studies mixed variable routes of administration and doses; and sample sizes were relatively small [18–20]. While these prior studies demonstrate the acceptability and feasibility of using CBD in conditions with symptomatic pain, ideal dosing for chronic pain is uncertain. Additionally, few studies have focused on the efficacy of CBD for chronic pain among vulnerable groups, including Veterans.

Thus, we developed a protocol to investigate whether CBD improves overall pain symptoms and quality of life among Veterans with chronic pain via a pragmatic, randomized, double-blinded, and placebo-controlled trial using CBD oral solution. Our objective is to examine whether CBD oral solution compared with placebo study medication is associated with changes in pain and related symptoms over 4 weeks of use. Our primary outcome is the Patient Global Impression of Change (PGIC), which is often used in trials for chronic pain [21], while our secondary outcomes include pain severity, pain interference, sleep, suicide ideation, and mood. As part of this study, we will also phenotype pain and investigate whether pain related symptoms (i.e., secondary outcomes) contribute to global improvement. Consistent with other clinical trials of CBD, we expect adverse events to be rare and minor [22].

## Methods

### Overview of trial design

This protocol details a pragmatic, randomized, double-blinded, and placebo-controlled trial to determine whether CBD improves overall pain symptoms and quality of life among Veterans with chronic pain. Enrolled participants will be randomized in a 1:1 ratio to receive either CBD oral solution or placebo study medication for 4 weeks (See Table 1 for more information). A Data Safety and Monitoring Board (DSMB) is overseeing trial conduct and safety, including recommendations regarding stopping the study for safety reasons. The study team will convene meetings with this independent board twice per year with ad hoc meetings in the event of unexpected or related serious adverse events.

**Table 1:** Intervention arms in a pragmatic trial and cannabidiol (CBD) to improve chronic pain symptoms among veterans

Agent (Source)	Precautions	Dose	Route	Schedule
Placebo (ElSohly Labs)	Consider taking with a meal to increase absorption	Days 1–28: suggest 0.2mL BID, up to 1.2mL per day	Oral liquid	Days 1–28: 0.2-0.6mL, taken BID, up to 1.2mL/day
Epidiolex (Jazz Pharmaceuticals)	Consider taking with a meal to increase absorption	Days 1–28: suggest 0.2mL BID, up to 1.2mL per day	Oral liquid	Days 1–28: 0.2-0.6mL, taken BID, up to 1.2mL/day

BID (bis in die): administered twice daily, the dosing schedule is a starting recommendation, so participants may flexibly modify their dosing regimen to another pattern if they choose

This trial will be conducted in collaboration with the University of Michigan Community Advisory Board for Veteran Pain Care and Research (Veteran CAB). The Veteran CAB includes Veterans, people who provide health care to Veterans, and representatives of Veterans’ groups. We will convene meetings between the study team and Veteran CAB 3–6 times per year. During these meetings, the research team will provide updates regarding study progress and next steps. Veteran CAB members have provided feedback on the trial, including choice of treatment, outcomes, recruitment materials, and approach. Over the course of the trial, the Veteran CAB will continue its critical input regarding study retention, interpretation of study findings, and dissemination of results.

**Fig. 1:**
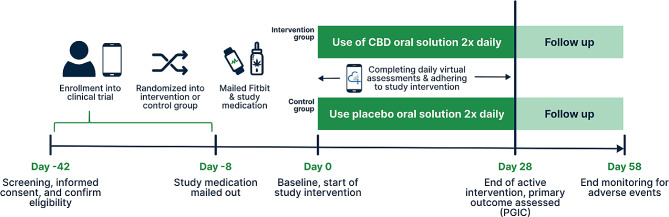
Study timeline for a pragmatic trial and cannabidiol (CBD) to improve chronic pain symptoms among Veterans. PGIC: Patient global impression of change

Figure 1 provides a timeline of study activities. As part of our pragmatic trial design, we will ship study materials to participants so they can complete all study activities remotely. Participants will enter data into MyDataHelps^®^ using a smartphone, computer, or similar device connected to the internet. MyDataHelps allows for the delivery of e-consent forms, syncing data from an actigraphy monitor watch (i.e., Fitbit^®^ or similar device) for the trial, and sending notifications that remind participants to complete assigned surveys.

As a double-blinded study, study investigators, the primary study statistician, and the study participants will be blinded to study group assignment during the trial. One study coordinator will be unblinded and work with the University of Michigan Research Pharmacy to assign participants. We will use unique identifiers to conceal allocation and an independent analyst will keep the assignment key confidential. Participants will remain blinded to allocation until the end of their trial participation. Study investigators and the primary statistician will remain blinded to allocation until the primary study analyses are complete. It is possible that randomization will be prematurely revealed to study team members and participants, for example, following adverse events as deemed by the DSMB.

### Intervention

In the active study arm, participants will self-administer CBD oral solution, Epidiolex^®^, which is manufactured by Jazz Pharmaceuticals. Epidiolex is a 100 mg/mL plant-derived CBD product approved by the FDA for seizures in Lennox-Gastaut and Dravet Syndrome [23–26]. ElSohly Labs will manufacture matching placebo study medication for participant self-administration. Jazz Pharmaceuticals and ElSohly Labs did not provide funding for this study, nor will they have any role in study design, conduct, data interpretation, or publication of results. The University of Michigan Research Pharmacy will dispense active or matching placebo oral solution in amber prescription bottles per the randomization code along with oral syringes and vial adapters to facilitate accurate dosing.

Initially, we will instruct participants to take 0.2mL of study medication in the morning and 0.2mL at night with a meal. Participants will then stay at this study medication dose or modify their regimen as desired up to 1.2mL (120 mg) per day and record their dosing in their daily diary. For example, some participants may wish to take 0.2-0.4mL three times per day, which would be acceptable given our pragmatic design. Participants will be told that taking study medication with a meal (especially a fatty meal) may increase absorption of CBD and lead to more sustained effects [27].

Since CBD products can be purchased over the counter or online, we believe it is critical to provide flexibility in dosing in the current trial to provide some structure while also pragmatically mimicking the naturalistic use environment. Further, we have selected our dosing window based on expert guidance from several published studies on cannabis and CBD dosing for chronic pain [28–32]. Given that CBD has been safely administered at much higher doses [22], we believe our dosing regimen will be well-tolerated.

### Ethics

This study was reviewed by the U.S. Food and Drug Administration (FDA), Federal Food, Drug, and Cosmetic Act for CBD and approved by the University of Michigan Institutional Review Board (IRB ID: HUM00231202).

### Trial registration

This study is registered in the ClinicalTrials.gov Database with ID: NCT06213233. Registered: 2 February 2024.

### Eligibility criteria

This trial is part of a larger program of work that includes a longitudinal registry of Veterans who have chronic pain and reside in a U.S. state where recreational cannabis is legal. Enrollment into this trial is limited to those who have consented to participate in the longitudinal registry and have completed at least 2 of 7 daily surveys each week as part of the registry for at least 4 consecutive weeks. The reason for this is twofold. First, it will help ensure that trial participants are able to successfully engage in remote study participation via the study app. Additionally, this will reduce the impact of regression to the mean in primary and secondary outcome measures [33].

#### Inclusion criteria

To be eligible for this trial, individuals must (a) be Armed Services Veterans aged 18 years or older; (b) report currently using cannabis for pain management or an interest in using cannabis for pain management; and (c) have moderate to severe chronic pain based on a self-report screening questionnaire (Table 2) that includes Pain Interference 4a short form items from the PROMIS-29 + 2 Profile v2.1 (PROPr) [34]. To allow for written informed consent and patient-reported outcome measures, participants must be able to read and speak English. To successfully complete study activities, participants must respond to study surveys on the MyDataHelps app, wear the study actigraphy monitor watch (Fitbit), be able to swallow the study medication, and adhere to the treatment regimen. Individuals of reproductive potential must not be pregnant or nursing at enrollment. Additionally, participants must agree to use birth control and must not donate sperm or ovum during study medication administration.

**Table 2:** Eligibility criteria for chronic pain in a pragmatic trial and cannabidiol (CBD) to improve chronic pain symptoms among veterans

Survey Items	Inclusion criteria
Over the last **3 months**, on average, how would you rate your pain on a scale of 0–10?	Must be ≥ 4
PROMIS^®^ 29 + 2 profile v2.1 (propr) Pain Interference In the past 7 days… 1. How much did pain interfere with your day to day activities? 2. How much did pain interfere with work around the home? 3. How much did pain interfere with your ability to participate in social activities? 4. How much did pain interfere with your household chores?	Must total ≥ 11 across the 4 items, which have the following response options and scores: Not at all: 1 A little bit: 2 Somewhat: 3 Quite a bit: 4 Very much: 5

#### Exclusion criteria

Participants will be excluded from this study if they are not an Armed Services Veteran or if they have a dishonorable discharge status. Inability to provide informed consent (e.g., cognitive impairment, unable to sufficiently communicate in English) will also be a basis for exclusion. Participants may not participate in any other clinical trials over the course of this study. Due to known medication interactions, participants will be excluded from the study if they currently use valproate or clobazam per self-report or medical records. Participants may be excluded from the study if they report any medical or psychiatric conditions that in the judgment of study personnel would preclude participation in this study (e.g., psychosis, suicide ideation; note that stable anxiety and depression are not exclusions). We will assess suicide ideation using the Positive and Negative Suicide Ideation questionnaire (PANSI) [35] with further risk assessment by the study psychiatrist. Participants may also be excluded if they have a serious or unstable hepatic disease (e.g., non-alcoholic fatty liver disease or liver cirrhosis); a major neurological disorder, such as dementia, Parkinson’s disease, cognitive impairment, epilepsy, and seizures; a current diagnosis of cancer; self-reported allergies to sesame oil, cannabis, or cannabinoids; or any impairment, activity, behavior, or situation that in the judgment of the study team would prevent satisfactory completion of the study protocol.

### Recruitment and retention

Our target sample size is 468 participants, which we selected to ensure sufficient power (i.e., at least 80%) to detect a relatively small Cohen’s d effect size (i.e., 0.30) for our primary outcome, PGIC, when assuming 25% attrition. We aim to recruit a study sample that is representative of the gender and racial/ethnic distribution of Veterans residing within the State of Michigan [36]. There are multiple resources available to us that will support recruitment for the proposed study. Based on medical chart review, we have identified 482 adult Veterans with chronic pain who have sought care through the University of Michigan Health System, and we will contact them with IRB approval. We will also recruit outside of the health system by contacting Veterans’ groups and organizations, attending Veteran-specific resource fairs, and sharing information at free community education events concerning chronic pain and the evidence-base for cannabis. We will supplement these recruitment approaches using the research volunteer registry, UMHealthResearch.com; targeted radio and social media ad campaigns; and word-of-mouth.

This study does not require in-person visits, which will support participant retention. We will compensate participants up to $60 for completing study-related surveys over 4 weeks. Additionally, participants will be able to keep the study Fitbit for personal use at the conclusion of the intervention.

### Trial procedures

We will pre-screen potential participants via telephone and using an online survey that includes questions regarding study eligibility criteria. We will preferentially offer this first contact, like all contacts and study meetings, via video conferencing or telephone. However, if participants express a strong desire for in-person visits and are willing to travel, the research team will accommodate in-person visits. This preliminary evaluation ensures the medical appropriateness of the participants to be enrolled in the study, which will be confirmed by study physicians. Next, a study team member will review the informed consent with those who pass pre-screening. Individuals will then be allotted 14 days to sign the informed consent remotely via the study app.

After completing informed consent, participants will have access to the study screening survey, which they must complete within 7 days. If participant responses do not reflect moderate to severe chronic pain defined as ≥ 4 on the 0–10 Numeric Rating Scale Pain scale and ≥ 11 on the Pain Interference assessment [34] (see Table 2), they will be ineligible and notified by a study team member. For those who meet the inclusion criteria, are female or intersex, are not permanently sterile or postmenopausal based on self-report, we will require an at-home urine pregnancy test. We will ship pregnancy tests to these individuals who must provide a picture of the test result to the study team prior to meeting with a study physician. Those who are not eligible due to a positive urine pregnancy result but meet other relevant eligibility criteria may re-screen one additional time in the future. Once study inclusion criteria are confirmed, participants will videoconference with a study physician. The study physician will review participant medical charts for eligibility criteria. If no exclusion criteria are identified, the participant is enrolled in the trial and ready to be randomized.

We are using permuted block randomization with the R package *blockrand* to randomize participants to receive either CBD oral solution or placebo study medication, stratified by sex assigned at birth (i.e., male, female, intersex) and varying block sizes of 2 and 4. Following randomization, the University of Michigan Research Pharmacy will ship the study medication to participants. After confirming receipt, study staff will provide an educational session that details how to administer the study medication and how to store it in a place that is only accessible to the participant. As part of this education for self-administration of study medication, participants will also receive pamphlet guides and videos. We will advise participants not to drive or operate machinery until they have gained sufficient experience on cannabidiol to personally gauge whether it adversely affects their ability to drive or operate machinery. We will also advise participants that concomitant use of alcohol with study drug may increase sedation and somnolence. During the trial, we will ask participants to refrain from starting any new treatment or pain therapy. Participants who already use cannabis products are asked to either refrain from or not increase their current use and to not try any new cannabis products during the trial. If a participant reports initiating a new treatment, the study team will assess the potential for increased risk (e.g., medication interactions).

Active study intervention will continue for 4 weeks unless it must be stopped sooner due to an unacceptable adverse event or participant withdrawal. After 4 weeks, we will ask participants to return remaining study medication via mail so that it can be measured and recorded. We will destroy returned study medication according to the institutional standard operating procedure for drug destruction. We will seek to follow participants for at least 30 days after active study intervention to monitor adverse events and collect outcome measures. After an adverse event in either study arm, participants will be followed until resolution or stabilization.

### Primary outcome

Our primary outcome measure will be the Patient Global Impression of Change (PGIC), a 1-item survey that measures patient perceptions of intervention success [37].

### Secondary outcome measures

We selected secondary outcomes in accordance with the Initiative on Methods, Measurement, and Pain Assessment in Clinical Trials (IMMPACT) recommendations on defining the clinical importance of treatment outcomes in studies of chronic pain [21, 38]. These measures are:

#### Pain interference

Measured using the Pain Interference 4a short form items from the PROMIS-29 + 2 Profile v2.1 (PROPr) [34]. We will assess changes in pain interference comparing placebo against CBD over 4 weeks from baseline to the end of intervention.

#### Pain intensity

We will ask participants to report their “worst pain” level on a 0–10 numerical rating scale via a brief daily questionnaire. To assess change in pain intensity over the 4 weeks of the intervention, we will compare the average of pain intensity for week 1 relative to the average of pain intensity for week 4.

#### Anxiety

We will measure anxiety using the anxiety items subscale from the PROMIS-29 + 2 Profile v2.1 (PROPr) [34] at baseline and week 4.

#### Sleep disturbance

We will measure sleep using the sleep disturbance items subscale from the PROMIS-29 + 2 Profile v2.1 (PROPr) [34] at baseline and week 4.

#### Suicide ideation

We will measure suicide ideation and vulnerability to suicide-related behavior with the PANSI [35] at baseline and week 4.

### Other measures

#### Demographics

We will use standardized case report forms to collect sex at birth, age, and race/ethnicity.

#### Daily assessments

We will ask participants to complete brief (< 5 min), daily questions for pain intensity, pain interference, and sleep quality using a 0–10 numeric rating scale, and record their daily cannabis use (i.e., product name, administration route, cannabinoid content, dose, timing, and side effects).

#### Fitbit data

Synced data from Fitbits will provide information that includes physical activity minutes, step count, sedentary time, sleep duration, and resting heart rate. We will ask participants to wear the Fitbits daily throughout the duration of the study for passive data collection.

#### Pain phenotypes

We will use the 2016 Fibromyalgia Survey Criteria to generate a continuous measure of nociplastic pain that ranges from 0 to 31 based on a self-report body map that assesses the number of painful sites as well as symptom severity for fatigue, cognitive problems, headache, and mood [7, 39, 40]. We will also measure neuropathic pain quality using painDETECT [41]. These combined measures will allow us to classify participants as having degrees of nociplastic pain and neuropathic pain, which may affect treatment response. We will collect these measures at baseline and week 4.

#### Social determinants of health

The screening survey will collect information about social determinants of health including employment status, household income, household size, financial strain, healthcare financial strain, transportation access, food insecurity, housing insecurity, social support, exposure to discrimination, and neighborhood cohesion and disorder [42–47].

### Safety and adverse events

We will ask participants to report adverse events on Day 1 (baseline), Day 7, Day 28 (week 4, end of treatment and primary outcome assessed), and Day 58 (end of follow up) via a brief questionnaire. Participants may also report adverse events as needed per institutional and FDA guidelines. If a participant reports pregnancy, we will withdraw them from the study. We will also ask participants about any new medications or supplements that they may have started taking, and whether they were diagnosed with a new medical condition. If this information suggests potential interactions with CBD, we will send participants for a blood draw in their local area to test liver function. Study investigators and physicians will determine whether a participant needs to be withdrawn from the study due to safety concerns on a case-by-case basis. Safety will also be a secondary outcome in this study.

### Data analysis plan

Primary analysis will use an intent-to-treat analysis and include all randomized participants regardless of their completion of the protocol. We will examine the distribution of variables and apply transformations if necessary. We will generate descriptive statistics such as means, medians, standard deviations, range, frequencies, and percentages overall and by intervention assignment, conditional on the scale of measurement.

The primary analysis will fit a linear regression model with the outcome of PGIC and main effect-coded binary predictor of CBD vs. placebo controlling for the stratification variable of sex at birth. We will use an alpha level of 0.05 for the main treatment effect of interest. We will include covariates such as age, race/ethnicity, baseline symptoms (e.g., anxiety, sleep disturbance), recruitment method (clinic vs. community engaged approach vs. social media/internet), concomitant medications, and pain phenotype (i.e., per the painDETECT and 2016 FM survey criteria) in secondary analyses. We will explore treatment effect modification by gender, CBD dose, and the above covariates by exploring treatment by covariate interactions using shrinkage methods. We will explore baseline sleep, anxiety, pain severity, and pain intensity, as possible mediators of the treatment effect with the primary outcome.

We will analyze secondary outcomes similarly using linear or generalized linear (depending on the outcome type) regression controlling for baseline value if recorded. To explore longitudinal changes, we will analyze weekly and daily outcomes using linear mixed effects models (with any transformation necessary). We will use mixed effects models with a random intercept for the participant and random slope of time and fixed effects of treatment arm, time, and the interactions between time and treatment to compare the treatment groups over time. In stratified analysis by treatment (CBD vs. placebo), we will assess the relationship between the primary and secondary outcomes and the average daily dose taken, and cumulative dose taken, separately.

Lastly, our exploratory analyses will consider daily assessments of pain-related symptoms, Fitbit data, and social determinants of health, e.g., whether activity levels or social risk factors moderate treatment efficacy.

### Missing data

We will implement weekly quality control checks to minimize missing data and monthly reports will summarize missingness. We will examine missingness by random group assignment to determine whether there is differential drop-out. Using two-sample t-tests, chi-square tests, and potentially their non-parametric equivalents, we will compare the characteristics of those who complete the study protocol and those who drop out or are lost to follow up. We will examine patterns of missing data and use multiple imputation methods for missing outcome measures under appropriate missingness assumptions. If there are any differences between completers and drop-outs, we will account for those variables in our imputation models.

## Discussion

Despite increasing preclinical evidence of the anti-inflammatory and analgesic potential of CBD [48], research in this area is limited and CBD is yet to be effectively integrated within chronic pain care protocols. Prior studies have demonstrated the feasibility of using CBD to treat conditions with symptomatic pain, but ideal dosing for chronic pain remains largely unexplored, especially in the context of different pain mechanism classifications. Additionally, few studies have examined the efficacy of CBD for chronic pain among Veterans, a group particularly vulnerable to the negative impacts of pain.

This protocol details the implementation of a randomized, double-blind, placebo-controlled, pragmatic clinical trial to examine whether CBD improves pain symptoms among Veterans with chronic pain. Once complete, this trial will be among the largest to date concerning the efficacy of CBD for chronic pain. Findings gained from this clinical trial will contribute to a greater knowledge of CBD’s analgesic potential and guide further research. Given the relative availability of CBD oral solution within health systems, our findings may underscore the potential of an accessible option for helping with the management of chronic pain among Veterans. Partnership with the Veteran CAB will increase input from the study population throughout the duration of the trial and facilitate the dissemination of study results to diverse audiences.

Thus far, the trial has not required changes to the protocol via an amendment. However, we anticipate that there may be challenges given the trial’s dependence on technology and remote participation. It may be difficult for participants to complete daily assessments depending on their access to internet and other factors. To ensure effective and timely communication with participants, we will use multiple approaches including email, text, and app notifications. We will also monitor study participation on a weekly basis. We anticipate knowledge gaps in the study population concerning CBD oral solutions that may impact recruitment efforts and require additional support from the study team as participants learn how to self-titrate the assigned study medication. As part of the trial enrollment process, a member of the study team will speak with participants to provide information and answer questions regarding study medication dosing and self-administration. We will also give participants paper and electronic copies of the Study Medication Guide, as well as a video demonstrating how to use the study medication. To increase compliance for return of study medication at the end of the trial, we will send participants pre-paid return labels and shipping materials.

## Data Availability

No datasets were generated or analysed during the current study.

## References

[1] Institute of Medicine: Relieving Pain in America: a blueprint for transforming Prevention, Care, Education, and Research. In. Washington, DC: National Academies; 2011. 22553896

[2] Nahin RL, Feinberg T, Kapos FP, Terman GW (2023). Estimated Rates of Incident and Persistent Chronic Pain among US adults, 2019–2020. JAMA Netw Open.

[3] Enkema MC, Hasin DS, Browne KC, Stohl M, Shmulewitz D, Fink DS, Olfson M, Martins SS, Bohnert KM, Sherman SE (2022). Pain, cannabis use, and physical and mental health indicators among veterans and nonveterans: results from the national epidemiologic survey on Alcohol and related Conditions-III. Pain.

[4] Qureshi AR, Patel M, Neumark S, Wang L, Couban RJ, Sadeghirad B, Bengizi A, Busse JW. Prevalence of chronic non-cancer pain among military veterans: a systematic review and meta-analysis of observational studies. BMJ Mil Health 2023. 10.1136/military-2023-002554 38124087

[5] Clauw DJ (2014). Fibromyalgia: a clinical review. JAMA.

[6] Finnerup NB, Attal N, Haroutounian S, McNicol E, Baron R, Dworkin RH, Gilron I, Haanpaa M, Hansson P, Jensen TS (2015). Pharmacotherapy for neuropathic pain in adults: a systematic review and meta-analysis. Lancet Neurol.

[7] Wolfe F, Fibromyalgianess (2009). Arthritis Rheum.

[8] Woolf CJ (2011). Central sensitization: implications for the diagnosis and treatment of pain. Pain.

[9] Yunus MB (2007). Role of central sensitization in symptoms beyond muscle pain, and the evaluation of a patient with widespread pain. Best Pract Res Clin Rheumatol.

[10] Kosek E, Cohen M, Baron R, Gebhart GF, Mico JA, Rice AS, Rief W, Sluka AK (2016). Do we need a third mechanistic descriptor for chronic pain states?. Pain.

[11] Harte SE, Harris RE, Clauw DJ (2018). The neurobiology of central sensitization. J Appl Biobehav Res.

[12] Fitzcharles MA, Cohen SP, Clauw DJ, Littlejohn G, Usui C, Häuser W (2021). Nociplastic pain: towards an understanding of prevalent pain conditions. Lancet.

[13] Malfait AM, Gallily R, Sumariwalla PF, Malik AS, Andreakos E, Mechoulam R, Feldmann M (2000). The nonpsychoactive cannabis constituent cannabidiol is an oral anti-arthritic therapeutic in murine collagen-induced arthritis. Proc Natl Acad Sci U S A.

[14] Hammell DC, Zhang LP, Ma F, Abshire SM, McIlwrath SL, Stinchcomb AL, Westlund KN (2016). Transdermal cannabidiol reduces inflammation and pain-related behaviours in a rat model of arthritis. Eur J Pain.

[15] Philpott HT, O’Brien M, McDougall JJ (2017). Attenuation of early phase inflammation by cannabidiol prevents pain and nerve damage in rat osteoarthritis. Pain.

[16] Costa B, Trovato AE, Comelli F, Giagnoni G, Colleoni M (2007). The non-psychoactive cannabis constituent cannabidiol is an orally effective therapeutic agent in rat chronic inflammatory and neuropathic pain. Eur J Pharmacol.

[17] De Gregorio D, McLaughlin RJ, Posa L, Ochoa-Sanchez R, Enns J, Lopez-Canul M, Aboud M, Maione S, Comai S, Gobbi G (2019). Cannabidiol modulates serotonergic transmission and reverses both allodynia and anxiety-like behavior in a model of neuropathic pain. Pain.

[18] Hunter D, Oldfield G, Tich N, Messenheimer J, Sebree T (2018). Synthetic transdermal cannabidiol for the treatment of knee pain due to osteoarthritis. Osteoarthr Cartil.

[19] Xu D, Cullen B, Tang M, Fang Y. The Effectiveness of Topical Cannabidiol Oil in Symptomatic Relief of Peripheral Neuropathy of the Lower Extremities. *Current pharmaceutical biotechnology* 2019. 10.2174/1389201020666191202111534 31793418

[20] Nitecka-Buchta A, Nowak-Wachol A, Wachol K, Walczynska-Dragon K, Olczyk P, Batoryna O, Kempa W, Baron S. Myorelaxant Effect of Transdermal Cannabidiol Application in patients with TMD: a Randomized, double-blind trial. J Clin Med 2019, 8(11). 10.3390/jcm8111886 PMC6912397 31698733

[21] Dworkin RH, Turk DC, Wyrwich KW, Beaton D, Cleeland CS, Farrar JT, Haythornthwaite JA, Jensen MP, Kerns RD, Ader DN (2008). Interpreting the clinical importance of treatment outcomes in chronic pain clinical trials: IMMPACT recommendations. J Pain.

[22] Iffland K, Grotenhermen F (2017). An update on Safety and Side effects of Cannabidiol: a review of Clinical Data and relevant Animal studies. Cannabis Cannabinoid Res.

[23] Devinsky O, Marsh E, Friedman D, Thiele E, Laux L, Sullivan J, Miller I, Flamini R, Wilfong A, Filloux F (2016). Cannabidiol in patients with treatment-resistant epilepsy: an open-label interventional trial. Lancet Neurol.

[24] Devinsky O, Cross JH, Laux L, Marsh E, Miller I, Nabbout R, Scheffer IE, Thiele EA, Wright S (2017). Cannabidiol in Dravet Syndrome Study G: Trial of Cannabidiol for drug-resistant seizures in the Dravet Syndrome. N Engl J Med.

[25] Devinsky O, Patel AD, Thiele EA, Wong MH, Appleton R, Harden CL, Greenwood S, Morrison G, Sommerville K, Group GPAS (2018). Randomized, dose-ranging safety trial of cannabidiol in Dravet syndrome. Neurology.

[26] Thiele EA, Marsh ED, French JA, Mazurkiewicz-Beldzinska M, Benbadis SR, Joshi C, Lyons PD, Taylor A, Roberts C, Sommerville K (2018). Cannabidiol in patients with seizures associated with Lennox-Gastaut syndrome (GWPCARE4): a randomised, double-blind, placebo-controlled phase 3 trial. Lancet.

[27] Silmore LH, Willmer AR, Capparelli EV, Rosania GR (2021). Food effects on the formulation, dosing, and administration of cannabidiol (CBD) in humans: a systematic review of clinical studies. Pharmacotherapy.

[28] MacCallum CA, Russo EB (2018). Practical considerations in medical cannabis administration and dosing. Eur J Intern Med.

[29] MacCallum CA, Eadie L, Barr AM, Boivin M, Lu S (2021). Practical strategies using medical Cannabis to reduce Harms Associated with Long Term Opioid Use in Chronic Pain. Front Pharmacol.

[30] Bhaskar A, Bell A, Boivin M, Briques W, Brown M, Clarke H, Cyr C, Eisenberg E, de Oliveira Silva RF, Frohlich E (2021). Consensus recommendations on dosing and administration of medical cannabis to treat chronic pain: results of a modified Delphi process. J Cannabis Res.

[31] Boehnke KF, Clauw DJ (2019). Brief commentary: cannabinoid dosing for chronic Pain Management. Ann Intern Med.

[32] Boehnke KF, Clauw DJ (2022). Cannabinoids for Chronic Pain: translating systematic review findings into clinical action. Ann Intern Med.

[33] Cummings JL, Tractenberg RE, Gamst A, Teri L, Masterman D, Thal LJ (2004). Regression to the mean: implications for clinical trials of psychotropic agents in dementia. Curr Alzheimer Res.

[34] PROMIS Profile 20 + 2. (PROPr) [http://www.healthmeasures.net/images/PROMIS/manuals/PROMIS_adult_profile_scoreing_manual.pdf].

[35] Muehlenkamp JJ, Gutierrez PM, Osman A, Barrios FX (2005). Validation of the positive and negative suicide ideation (PANSI) inventory in a diverse sample of young adults. J Clin Psychol.

[36] MVAA: MVAA 2021 Annual Report. In. Michigan.gov/MVAA. 2021: 20.

[37] Guy W (1976). ECDEU assessment manual for psychopharmacology.

[38] Dworkin RH, Turk DC, Farrar JT, Haythornthwaite JA, Jensen MP, Katz NP, Kerns RD, Stucki G, Allen RR, Bellamy N (2005). Core outcome measures for chronic pain clinical trials: IMMPACT recommendations. Pain.

[39] Brummett CM, Bakshi RR, Goesling J, Leung D, Moser SE, Zollars JW, Williams DA, Clauw DJ, Hassett AL (2016). Preliminary validation of the Michigan Body Map. Pain.

[40] Wolfe F, Clauw DJ, Fitzcharles MA, Goldenberg DL, Häuser W, Katz RS, Mease P, Russell AS, Russell IJ, Winfield JB (2011). Fibromyalgia criteria and severity scales for clinical and epidemiological studies: a modification of the ACR Preliminary Diagnostic Criteria for Fibromyalgia. J Rheumatol.

[41] Freynhagen R, Baron R, Gockel U, Tolle TR (2006). painDETECT: a new screening questionnaire to identify neuropathic components in patients with back pain. Curr Med Res Opin.

[42] Gundersen C, Engelhard EE, Crumbaugh AS, Seligman HK (2017). Brief assessment of food insecurity accurately identifies high-risk US adults. Public Health Nutr.

[43] Andrea SB, Siegel SA, Teo AR (2016). Social Support and Health Service Use in depressed adults: findings from the National Health and Nutrition Examination Survey. Gen Hosp Psychiatry.

[44] Williams DR, Yan Y, Jackson JS, Anderson NB (1997). Racial Differences in Physical and Mental Health: Socio-economic status, stress and discrimination. J Health Psychol.

[45] Robinette JW, Charles ST, Gruenewald TL (2018). Neighborhood cohesion, neighborhood disorder, and cardiometabolic risk. Soc Sci Med.

[46] Mendes de Leon CF, Cagney KA, Bienias JL, Barnes LL, Skarupski KA, Scherr PA, Evans DA (2009). Neighborhood social cohesion and disorder in relation to walking in community-dwelling older adults: a multilevel analysis. J Aging Health.

[47] Sokol RL, Mehdipanah R, Bess K, Mohammed L, Miller AL (2021). When families do not request help: assessing a Social determinants of Health Screening Tool in Practice. J Pediatr Health Care.

[48] Finn DP, Haroutounian S, Hohmann AG, Krane E, Soliman N, Rice ASC (2021). Cannabinoids, the endocannabinoid system, and pain: a review of preclinical studies. Pain.

